# Construction of a diagnostic prediction model for ischemic stroke using lactylation-related genes

**DOI:** 10.3389/fnmol.2025.1672374

**Published:** 2025-11-25

**Authors:** Jierong Mo, Deyuan Ning, Shuyi Chen, Tianen Zhou, Peiyi Liu, Zhiquan Li, Wenmin Tang, Lianshan Guo, Zhiyong Lin, Ran Zhan, Jun Jiang, Xu Li

**Affiliations:** 1 Department of Emergency, Nanfang Hospital, Southern Medical University, Guangzhou, Guangdong, China; 2 Department of Emergency, The First People's Hospital of Foshan (Foshan Hospital Affiliated to Southern University of Science and Technology), School of Medicine, Southern University of Science and Technology, Guangzhou, Guangdong, China; 3 Department of Blood Transfusion, The First People's Hospital of Foshan (Foshan Hospital Affiliated to Southern University of Science and Technology), School of Medicine, Southern University of Science and Technology, Guangzhou, Guangdong, China

**Keywords:** lactylation, immune infiltration, machine learning, diagnostic prediction models, ischemic stroke

## Abstract

Ischemic stroke (IS) represents the leading global cause of acquired neurological disability and vascular-related mortality. However, diagnostic challenges persist in cases with atypical presentations. Lactylation modification exerts critical regulatory roles in disease pathogenesis and progression, and thus positioning as a potential diagnostic biomarker. We utilized weighted gene co-expression network analysis (WGCNA), gene ontology (GO)and Kyoto Encyclopedia of Genes and Genomes (KEGG), immune infiltration assessment, consensus clustering (via ConsensusClusterPlus), and multiple machine learning algorithms—including random forest (RF), support vector machine (SVM), neural network (NM), and generalized linear models (GLMs)—along with real-time-quantitative polymerase chain reaction (RT-qPCR) and western blot validation, to analyze gene expression omnibus (GEO) datasets. Our findings indicate that immune infiltration may play an important role in IS, with neutrophils and T cell receptor signaling pathway emerging as the most important immune cells and signaling pathway, respectively. Six hub genes, namely SLC2A3, NDUFB11, GTPBP3, SLC16A3, PUS1, and GRN, were identified and verified through RT-qPCR and the western blot. Surprisingly, the area under the curve (AUC) of the prediction model reached 0.968, with a 95% confidence interval ranging from 0.928 to 1. Extensive validation using multiple external GEO datasets confirmed the accuracy of the prediction model in five independent datasets. Furthermore, we observed that different concentrations of lactate could further suppress the proliferation of nerve cells following oxygen–glucose deprivation/reperfusion (OGD/R). This study provides a new diagnostic strategy for the early diagnosis of IS through the established diagnostic prediction model.

## 1 Introduction

Globally, among non-communicable diseases, stroke ranks as the second leading cause of death and the third leading cause of death and disability. The mortality and disability burden of stroke is particularly severe in low- and middle-income countries. From an economic perspective, the global cost of stroke is estimated to exceed $890 billion (0.66% of global GDP; Feigin et al., 2025). These statistics underscore the substantial threat stroke poses to human life and socioeconomic security. Ischemic stroke (IS) constitutes the predominant subtype of stroke. In Italy, for instance, IS accounts for 67.3–82.6% of cases, while hemorrhagic stroke represents 9.9–19.6% (Sacco et al., 2011). Acute IS typically arises from cerebral blood flow interruption, which may be caused by *in situ* arterial thrombosis, embolism originating from extracranial thrombi, or cerebral hypoperfusion due to other etiologies (Yu et al., 2023). Clinical evidence has demonstrated that early diagnosis and timely initiation of endovascular therapies for occluded artery recanalization can reduce disability and mortality in IS patients. Specifically, each 10-min delay in door-to-needle time reduces the probability of achieving favorable functional outcomes by 10% (Nguyen et al., 2024; Hilkens et al., 2024). Current early diagnosis of IS primarily relies on clinical evaluation and neuroimaging. However, in emergency departments, false-positive stroke diagnoses account for 30–43% of all suspected cases, while the false-negative rate reaches 28% among confirmed stroke patients. Diagnostic challenges are particularly prominent in cases presenting with atypical symptoms, such as visual disturbances, dizziness, vertigo, ataxia, or headache. Moreover, diagnostic accuracy is influenced by subjective factors: Approximately, 20–50% of acute suspected stroke diagnoses depend on whether patients are assessed by emergency physicians or stroke specialists (Helboe et al., 2023). In terms of imaging, acute stroke cannot be detected via computed tomography (CT) in the early disease stages, as CT findings may appear normal during this period (Akhtar et al., 2021). These limitations highlight the critical need to identify potential biomarkers and develop diagnostic models for early detection of IS.

Lactate, the primary end product of glycolysis under hypoxic conditions, has long been regarded as a metabolic waste product. However, emerging evidence in recent years has redefined the role of lactate, demonstrating that lactate induces a novel post-translational modification, lactylation, which exerts critical regulatory effects on the pathogenesis and progression of various diseases (Fan et al., 2023). Notably, both the abundance of and functional significance of lactate in the brain have been well established. Studies reveal that lactate in the brain is predominantly derived from glucose glycolysis in astrocytes and serves as an essential energy substrate for neuronal metabolism (Newman et al., 2011). Furthermore, lactate participates in intercellular communication by shuttling between adjacent cells or entering systemic circulation and exerting essential regulatory effects on cerebral blood flow, memory formation, neural function, neuroregeneration, and brain energy metabolism (Castro and Potente, 2022; Li et al., 2023). However, cerebral lactate can aggravate ischemic brain injury by promoting protein lactylation in IS (Xiong et al., 2024). Elevated lactate levels observed after IS enhance protein lactylation. Mechanistically, microglial SMEK1 ameliorates cerebral ischemia–reperfusion injury by modulating H3K9 lactylation (Si et al., 2024). Therefore, lactylation plays a pivotal pathophysiological role in IS, and the identification of its key regulators may be instrumental in development of early diagnostic biomarkers for IS.

The intense inflammatory response following IS exacerbates cerebral damage and impairs neurological function. The pathogenic mechanisms involve oxidative stress, upregulated matrix metalloproteinase production, microglial activation, and the infiltration of peripheral immune cells into ischemic lesions (Candelario-Jalil et al., 2022). Notably, hematogenous immune components—including monocytes, neutrophils, and T lymphocytes—critically mediate blood–brain barrier (BBB) disruption, a pivotal pathophysiological process underlying post-IS malignant cerebral edema and hemorrhagic transformation (Qiu et al., 2021). These cascades position immunoinflammatory pathways as potential therapeutic targets. In addition, emerging evidence reveals intricate crosstalk between lactylation regulation and immune infiltration. For example, lactylation-driven epigenetic reprogramming modulates diverse immune cell subsets and orchestrates disease progression in malignancies, inflammatory bowel disease, and rheumatoid arthritis (Hao et al., 2024; Wu et al., 2024; Liu et al., 2025). Hence, systematic investigation of lactylation-associated genetic regulators and their interplay with post-IS neuroimmune networks represents an urgent research priority.

In this investigation, we adopted an integrated bioinformatic interrogation strategy that combines differential expression analysis (DEGs), weighted gene co-expression network analysis (WGCNA), and lactylation-related genes (LRGs) profiling on gene expression omnibus (GEO) datasets, identifying 6 hub LRGs associated with IS. Molecular subtyping through consensusclustering revealed distinct pathophenotypes. Multiple immune infiltration scoring methods (CIBERSORT, MCP-counter, xCell, and quanTIseq) demonstrated neutrophil hyperactivation in IS; furthermore, the correlation between six hub genes and neutrophils was analyzed via correlation analysis. Subtype analysis was conducted to identify the differences in enrichment pathways and inflammatory infiltration. In addition, a variety of machine learning algorithms were applied based on six hub genes, and the GLM model with the optimal performance was selected for nomogram construction. Subsequently, the performance of the prediction model was validated using five external datasets. Finally, this study provides new insights into the early diagnosis of IS and reveals the potential associations between lactylation, neutrophils, and IS.

## 2 Materials and methods

### 2.1 Data acquisition

Gene expression series matrices of IS-related datasets were obtained from GEO database.^1^ The R package “GEO query” was used for acute IS dataset processing and gene symbol conversion. The GSE16561 dataset served as the primary analysis object, which contained peripheral blood mRNA gene expression information of 39 IS patients (diagnosed as IS by MRI) and 24 non-stroke controls. Subsequently, five independent IS-related datasets were used for external validation after the establishment of the prediction model. In addition, a total of 394 lactate-related genes (LRGs) were retrieved and downloaded from the MSigDB database (accessed on March 17, 2025).^2^

### 2.2 Identification of DEGs and enrichment analysis of gene ontology and Kyoto encyclopedia of genes and genomes

To identify DEGs between the IS and control cohorts, we conducted DEGs using the limma package (v3.40.6) in R, applying screening thresholds of |fold change(FC)| > 1.5 and *p*-value <0.05 (Liu et al., 2021). The resulting DEGs were visualized using volcano plots and heatmaps. Subsequently, systematic functional annotation of DEGs was conducted using GO and KEGG enrichment analyses. Briefly, the org. Hs.eg.db package (v3.1.0) was used as the gene annotation background, while KEGG pathway annotations were retrieved via the KEGG REST API. Both enrichment analyses were performed using the clusterProfiler package (v3.14.3). A *p*-value of < 0.05 and an false discovery rate (FDR) of< 0.1 were considered statistically significant.

### 2.3 Immune infiltration profiling

To comprehensively characterize alterations in the immune microenvironment of IS, four immune scoring systems—CIBERSORT, MCP-counter, xCell, and quanTIseq—were applied to calculate the immune cell infiltration score for each sample based on the expression profile via the R software package “IOBR” to mitigate method-specific biases (Chen et al., 2018; Wei et al., 2023). Statistical comparison of immune cell infiltration scores between the IS and control groups was performed using non-parametric Kruskal–Wallis tests. Immune cell subtypes demonstrating consistent differential patterns (*p* < 0.05) across all four algorithms were identified through intersection analysis.

### 2.4 Weighted correlation network analysis

WGCNA is a systems biology approach that analyzes patterns of gene association between different samples and can be used to identify gene sets with high covariation and to identify candidate biomarker genes or therapeutic targets. We performed WGCNA analysis on gene expression matrix data and calculated the median absolute deviation of each gene separately. The top 85% of the smallest median absolute deviation genes were removed, and the scale-free co-expression network was constructed with WGCNA. The minimum size of gene tree (genome) was 30, the sensitivity was 3, and the module merging threshold was 0.4. The average linkage hierarchical clustering was performed based on the dissimilarity measure derived from the topological overlap matrix. Next, Pearson correlation analysis was used to calculate the correlation between module membership (MM) and gene significance (GS). The key genes of WGCNA module were identified using a module membership (MM) threshold of 0.6 and a gene significance (GS) threshold of 0.4 (Langfelder and Horvath, 2008). Finally, hub genes associated with IS were screened by identifying the intersection of WGCNA key genes, DEGs, and LRGs.

### 2.5 Gene set enrichment analysis

GSEA was employed to assess the tendency of genes in a predefined gene set to be distributed in a list of genes ranked by phenotypic correlation, thus judging their contribution to the phenotype. Briefly, GSEA software (version 3.0) was downloaded from the GSEA website.^3^ Samples were divided into high- and low-expression groups based on the expression levels of hub genes. Subsequently, we downloaded the c2.cp.kegg.v7.4.symbols.gmt from MSigDB^4^ to evaluate related pathways and molecular mechanisms (Subramanian et al., 2005). A *p*-value of < 0.05 were considered statistically significant.

### 2.6 Construction of miRNA–mRNA network

In IS, miRNA–mRNA regulatory network serves as a key regulatory pathway mediating the effects of multiple upstream molecules (e.g., LncRNA and CircRNA; Kong et al., 2021). To explore the potential upstream miRNAs of the hub genes, we predicted candidate upstream miRNAs using Targetscan 8.0.^5^ The top 10 miRNAs were selected based on the context++ score, and the miRNA–mRNA regulatory network was constructed using Cytoscape (version 3.7.2).

### 2.7 ConsensusClusterPlus and subtype enrichment analysis

To explore the subtype analysis of IS, we used the expression matrix of six hub genes to classify 39 IS samples into subtypes. ConsensusClusterPlus was employed as a class discovery tool with confidence assessment and item tracking. Agglomerative pam clustering was performed using 1-pearson correlation distances, with 80% resampling of the samples for 1,000 repetitions. Finally, the empirical cumulative distribution function plot was used to determine the optimal cluster number (Wilkerson and Hayes, 2010). After subtypes of IS were classified, immune cell infiltration scores of two subtypes were calculated using four algorithms—CIBERSORT, MCPCounter, xCell, and quanTIseq. Finally, GO and KEGG enrichment analyses were performed using the expression matrix of DEGs of subtypes.

### 2.8 Construction and internal verification of machine learning prediction models

To evaluate the diagnostic predictive value of the six hub genes for IS, we constructed disease prediction models using four machine learning algorithms—RM, SVM, NN, and GLM (Zhang et al., 2024). Approximately, 70% of the GSE16561 dataset was used as a training set and 30% as a validation set. In RM, the largest tree in the forest is 50. Besides, SVM parameters are set as follows: penalty coefficient = 1.0, maximum number of iterations = 2,000, and kernel function = rbf. In addition, NN parameters are set as follows: penalty coefficient = 1.0, maximum number of iterations = 10,000, and activation function = relu. Moreover, in GLM, the “lrm” function of the “rms” package was used to build a predicted model, and the “nomogram” function was used to build a nomogram diagram, and the “calibrate” function was used to evaluate the consistency between the predicted probability and the actual probability, and the “roc” and “auc” functions are used to calculate the receiver operating characteristic (ROC) curve and area under the curve (AUC) values. Finally, the “decision curve” function of the “rmda” package was used to formulate the fitted risk value.

### 2.9 External validation of the predictive model

To verify the accuracy of the IS prediction model, another five GEO datasets were used for external validation. The expression matrix of the six hub genes or partial hub genes (note: some of the six hub genes were not detected in certain datasets.) in each dataset were used to calculate ROC curve, using the parameters and criteria of the previously constructed GLM. The detailed information of these GEO datasets is shown in Table 1 as follows.

**Table 1:** IS-related datasets with a sample size greater than 30 in the GEO database.

Dataset	GEO ID	Platform	Samples (Total)	Samples (Stroke)	Samples (Control)	Public data
1	GSE119121	GPL6247	47	8	39	8/29/2018
2	GSE58294	GPL570	92	69	23	9/2/2014
3	GSE36010	GPL7294	44	24	20	12/1/2012
4	GSE23160	GPL6885	32	24	8	1/10/2011
5	GSE22255	GPL570	40	20	20	12/31/2011

### 2.10 RT–qPCR

PC12 cells, a commonly used neuronal cell line, was widely used in the studies investigating neurological diseases. The OGD/R model of PC12 cell was used to simulate IS *in vitro*. The expression level of hub genes following different durations of OGD/R were verified via RT–qPCR. Briefly, reverse transcription of RNA was performed according to the instructions of All-In-One 5X RT MasterMix (abm, Canada), and qPCR was performed using BlasTaq™ 2X qPCR Master Mix (abm, Canada). Primer sequences for the hub genes are listed in Table 2.

**Table 2:** Primer sequences of hub genes.

Gene	Sequence	
SLC2A3	F: 5’ATGTTGGCCAGTCAAGTTCC3’	R: 5’CTGTCACCTCTGGGAGCAG3’
GRN	F: 5’CACTGTCCTGATGGCTACTCTTG3’	R: 5’CTACCAGGACACTGGACAGCAC3’
SLC16A3	F: 5’CCAGGCCCACGGCAGGTTTC3’	R: 5’GCCACCGTAGTCACTGGCCG3’
NDUFB11	F: 5’CTTCCAGGGCTGTAATCGC3’	R: 5’GGTTCTTCGCGTAGAGGTTT3’
GTPBP3	F: 5’CTGAACTGGCTGCAGTGTGTG3’	R: 5’CCTGTGAGGTGGCTTAGCTG3’
PUS1	F: 5’CGCACAGACAAGGGTGTGTC3’	R: 5’TCCTGTACATCCCGGTCCTT3’

### 2.11 Cell proliferation

The Cell Counting Kit 8 (CCK8; Biosharp, China) was used to assess neuronal cells proliferation according to the manufacturer’s instructions. The neuronal cells were treated with lactate at different concentrations (10 mM, 30 mM, and 50 mM) after oxygen–glucose deprivation/reoxygenation (OGD/R).

### 2.12 Western blot

The PC12 cells were treated with oxygen/glucose deprivation for 6 h and reperfusion for 24 h. The RIPA lysis buffer (Biosharp company, China) was used to extract the total proteins of the cells. Then, SDS-PAGE was used to separate different proteins. After electrophoresis, the proteins were transferred onto a PVDF membrane (EMD Millipore, Billerica, MA, United States). The proteins were incubated with primary antibodies of SLC2A3 (ProMab, Cat P22941, 1:1000), SLC16A3 (ProMab, Cat P12175, 1:1000), NDUFB11 (ProMab, Cat P21725, 1:1000), PUS1 (ProMab, Cat P21909, 1:1000), GTPBP3 (FineTest, Cat FNab03721, 1:1000), GRN (FineTest, Cat FNab03632, 1:1000), and secondary antibodies of anti-rabbit IgG HRP-linked antibody (Cell Signaling, United States). Finally, the images were developed using an exposure device (MiniChemi 910, SAGE CREATION, China).

## 3 Results

### 3.1 DEGs and enrichment analysis

**Figure 1:**
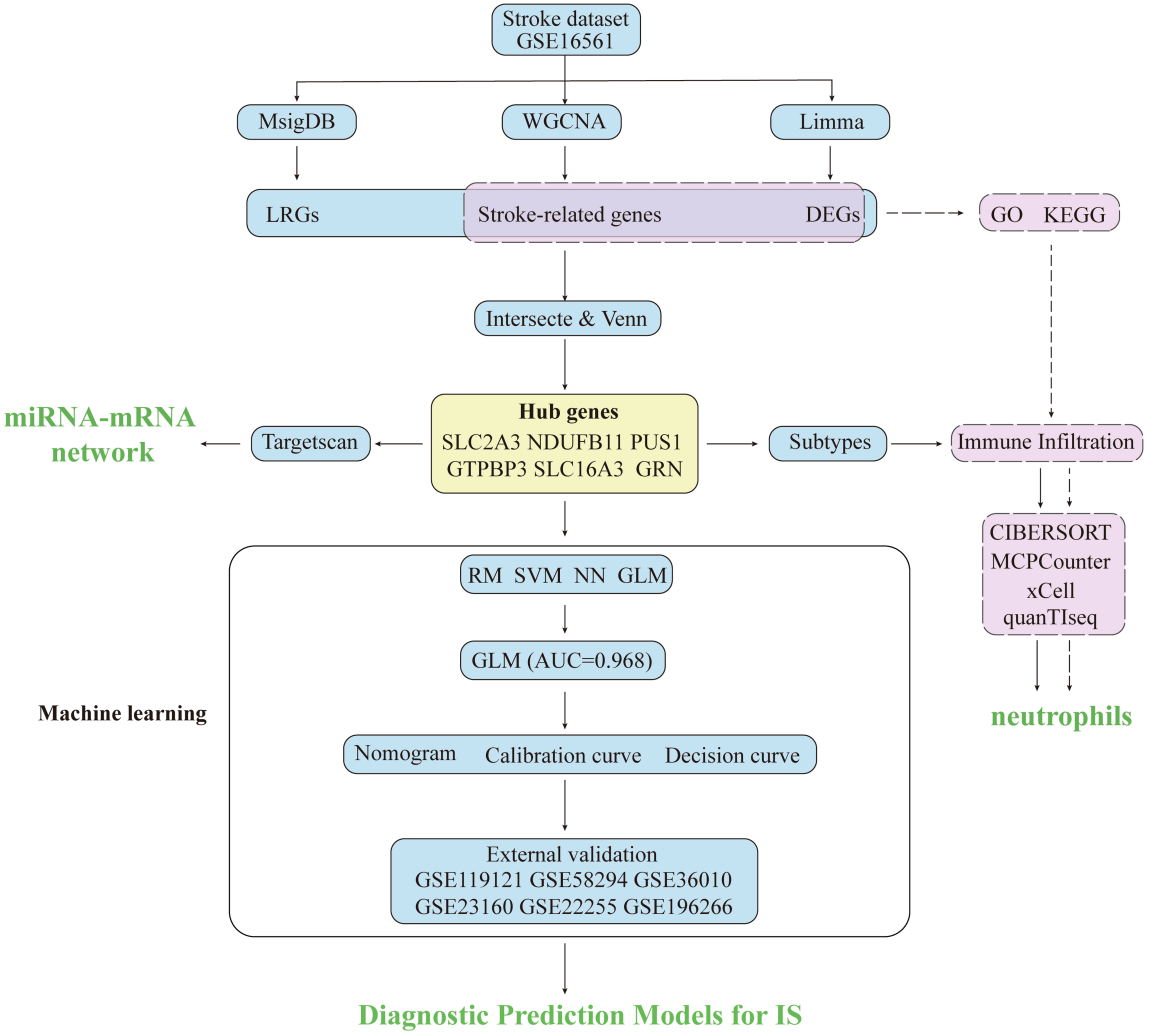
The main workflow of this study.

**Figure 2:**
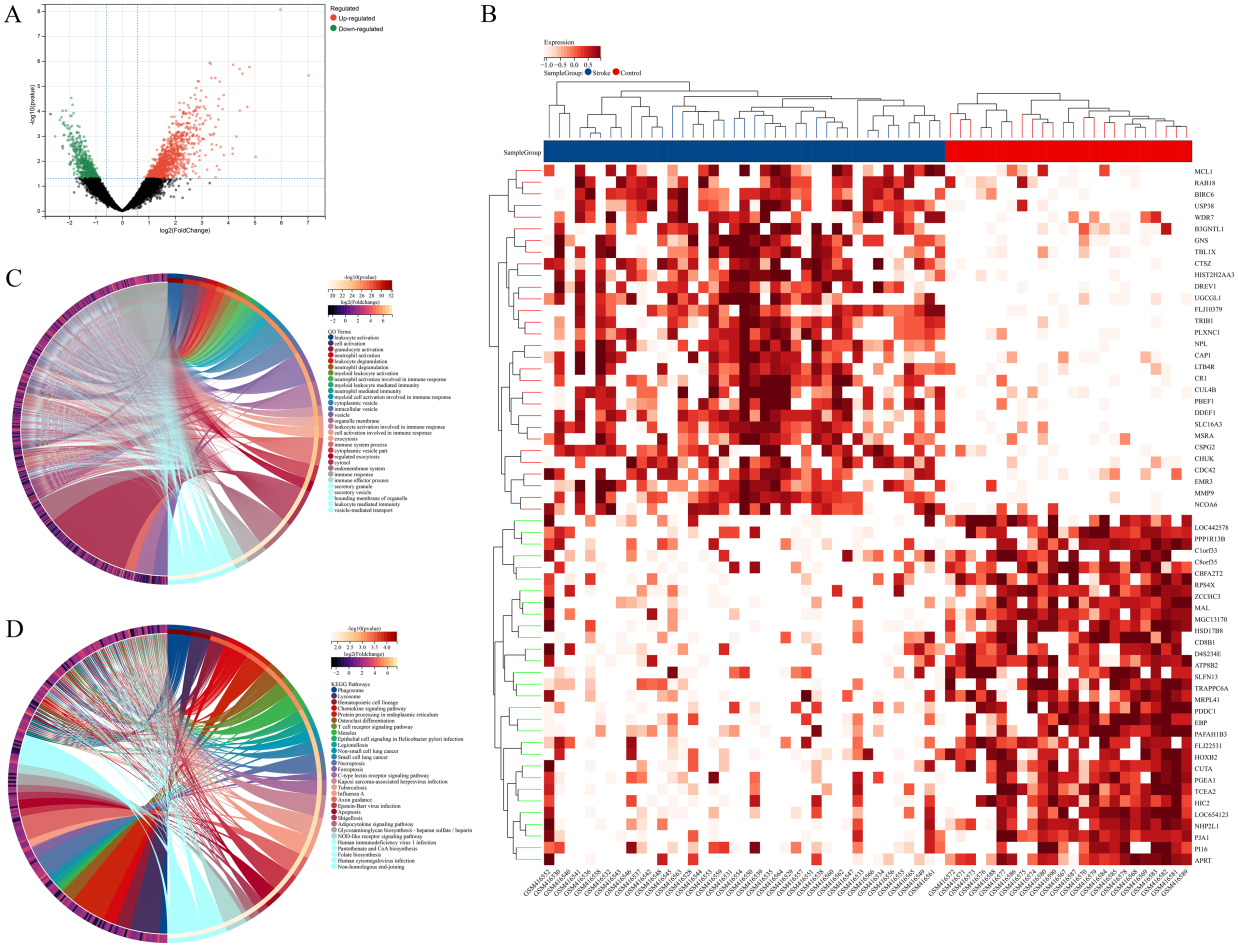
**(A)** Volcanic maps showed DEGs between the ischemic stroke group and the control group. **(B)** Heat map showed the top 30 upregulated genes and top 30 downregulated genes between the ischemic stroke group and the control group. **(C)** The result of DEGs enrichment analysis in GO. **(D)** The result of DEGs enrichment analysis in KEGG.

The main workflow of our study was shown in Figure 1. After screening with the threshold of |FC| > 1.5 and a *p*-value of < 0.05, a total of 1,071 upregulated genes and 564 downregulated genes were identified, with the results visualized in a volcano diagram (Figure 2A). Further analysis revealed 59 upregulated genes and 10 downregulated genes with a false discovery rate (FDR) of < 0.05; this observation may be attributed to the large sample size. A heat map was generated to display the top 30 genes that were most significantly upregulated and downregulated (Figure 2B). To explore the potential functions and signaling pathways of DEGs, GO and KEGG enrichment analyses were conducted. The top 30 most significant results of GO enrichment analysis were shown in Figure 2C; most of them were related to immune response; in particular, the top 10 terms were exclusively related to immune response (e.g., neutrophils, leukocyte, myeloid cells, and so on). The top 30 most significant results of KEGG enrichment analysis were presented in Figure 2D, including lysosome, chemokine signaling pathway, T cell receptor signaling pathway, necroptosis, ferroptosis, axon guidance, and apoptosis and so on.

### 3.2 Analysis of immune infiltration

**Figure 3:**
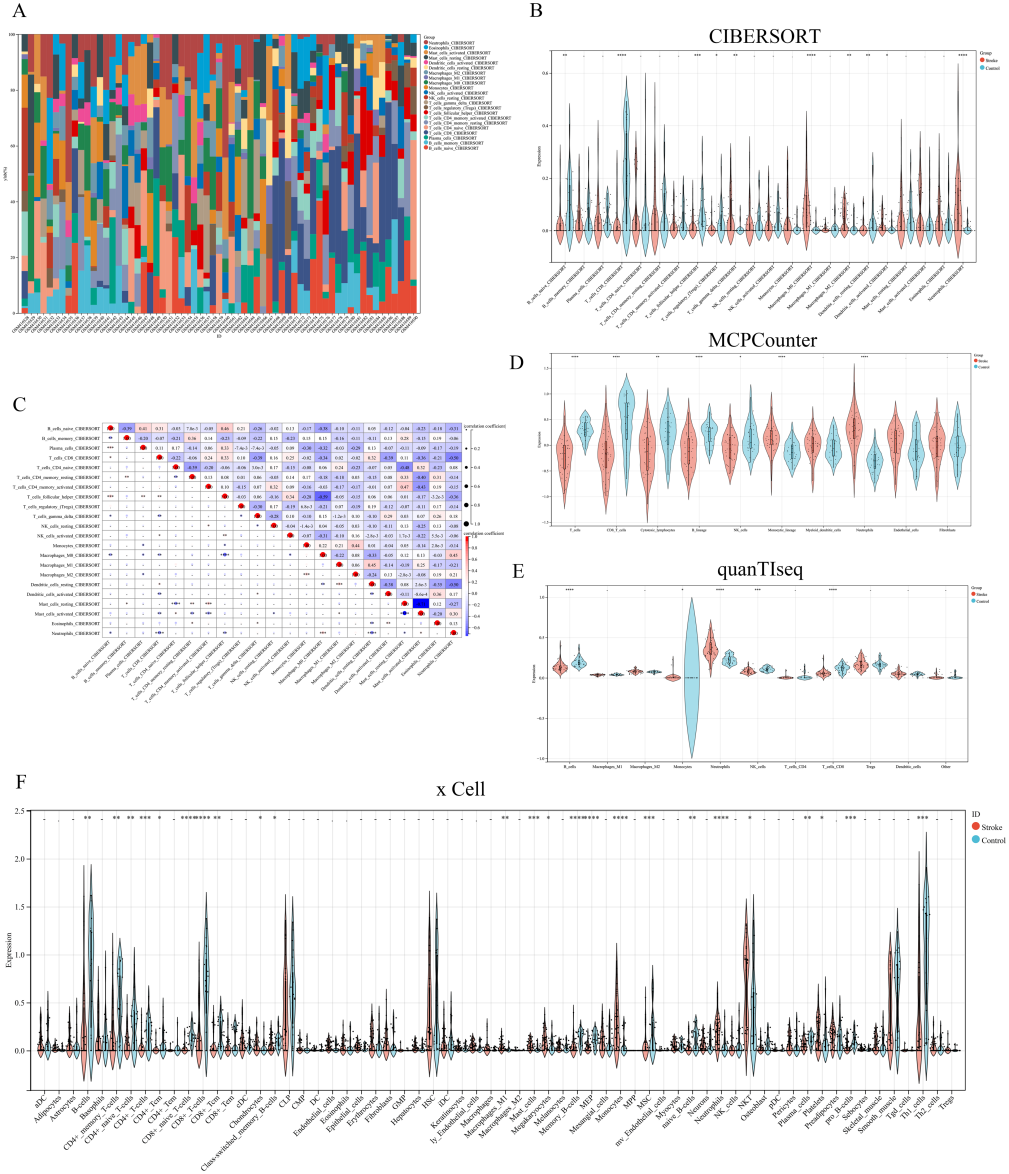
**(A)** Immune cell infiltration across samples in dataset. **(B)** The difference of immune infiltration between the ischemic stroke group and the control group was evaluated by CIBERSORT. **(C)** The correlation between immune cells. **(D–F)** The difference of immune infiltration between the ischemic stroke group and the control group was evaluated by MCPCounter, quanTIseq, and xCell, separately. *p < 0.05, **p < 0.01, ***p < 0.001, ****p < 0.0001.

Since the enrichment analysis of DEGs were primarily focused on immune response and inflammation constitutes a key factor contributing to brain function impairment in IS, we further performed immune infiltration analysis using multiple immune scoring systems. The result of CIBERSORT showed that B cells naive, T cells CD8, T cells follicular helper, T cells regulatory (Tregs), T cells gamma delta, macrophages M0, macrophages M2, dendritic cells resting, dendritic cells activated, and neutrophils were significantly different between the IS and control groups (Figures 3A–C). In addition, the results of MCPCounter, quanTIseq, and xCell were shown in Figures 3D–F, respectively. It was noteworthy that neutrophils were the only immune cell type exhibiting significantly higher infiltration levels in the IS group compared with the control group across all four algorithms.

### 3.3 Identification the module genes of IS via WGCNA

**Figure 4:**
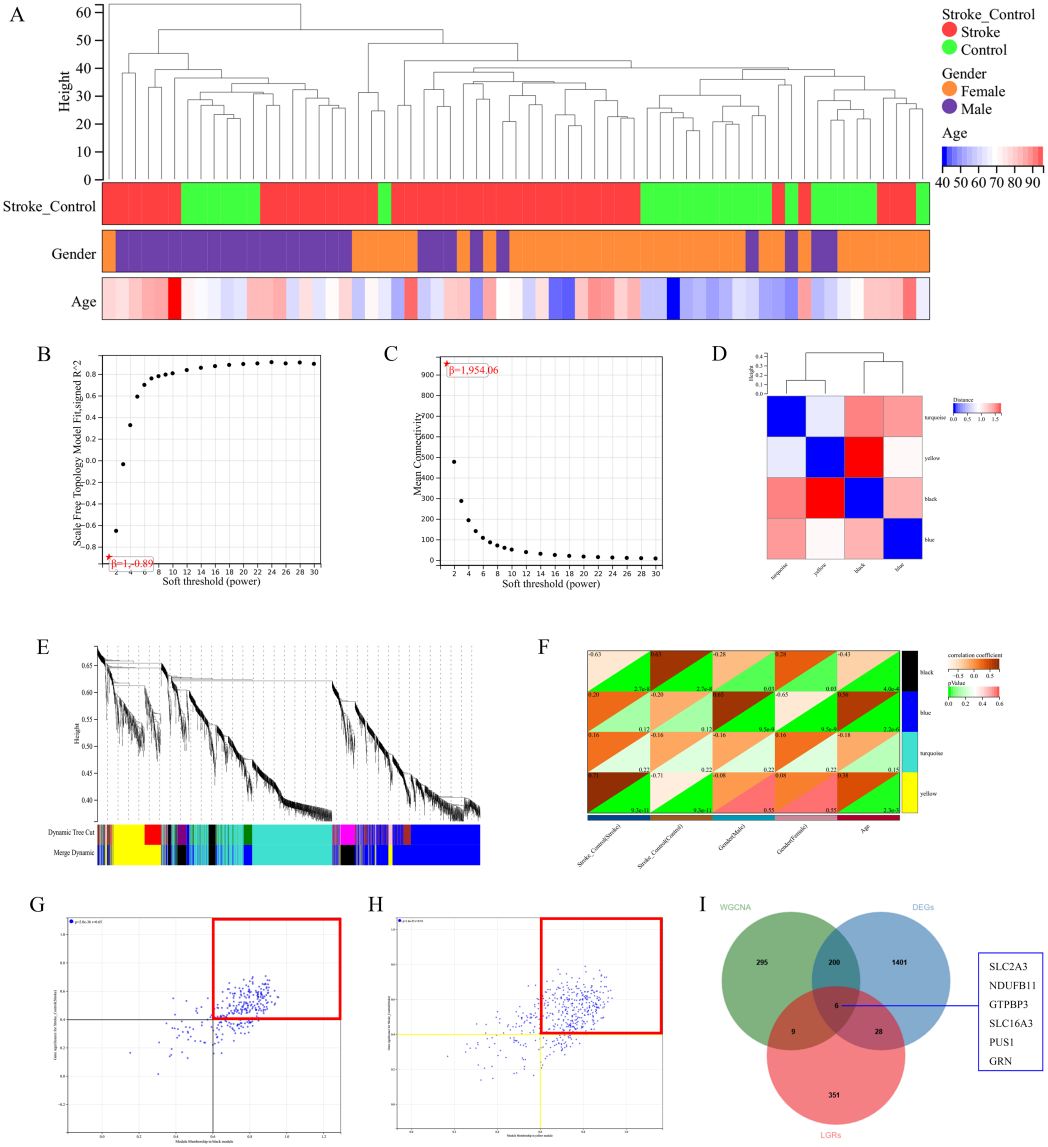
**(A)** Clustering results of samples and clinical features in WGCNA. **(B,C)** Soft threshold of WGCNA. **(D,E)** The result of Module feature vector cluster analysis and genes cluster analysis, separately. **(F)** A heat map of correlation between cluster modules and phenotypes. **(G,H)** Scatter diagram of the correlation between GS and MM. **(I)** Intersection analysis of DEGs, WGCNA, and LRGs. A *p*-value of < 0.05 was considered statistically significant.

**Figure 5:**
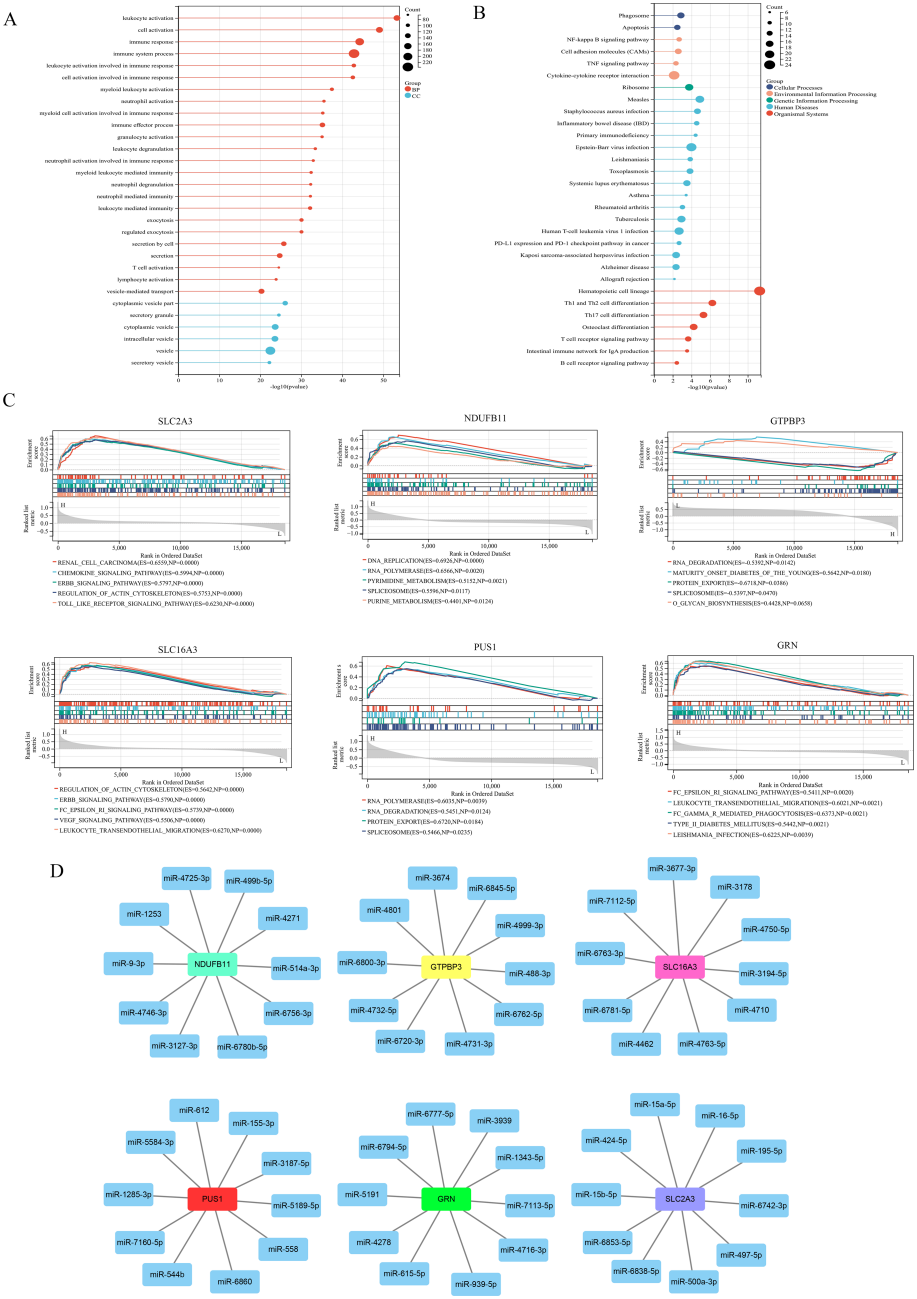
**(A)** GO enrichment analysis of stroke-related WGCNA module genes. **(B)** KEGG enrichment analysis of stroke-related WGCNA module genes. **(C)** GSEA analysis of hub genes. **(D)** The predicted network of miRNA–mRNA.

Genome modules with similar functions were identified via WGCNA analysis, and the results of sample clustering results were presented in Figure 4A. *β* serves as a soft-thresholding parameter that emphasizes strong correlations between genes while penalizing weak ones. The results of soft threshold were shown in Figures 4B,C. The results of module clustering were shown in Figures 4D,E, with a total of four modules (black, blue, turquoise, and yellow) identified. In addition, the correlation between modules and phenotype showed that the black module and the yellow module were correlated with stroke, the black module and the blue module were correlated with sex, and all modules except the turquoise modules were correlated with age (Figure 4F). Furthermore, we identified IS-associated module genes (Figures 4G,H) by setting the thresholds for GS and MM to 0.4 and 0.6, respectively. Next, we took the intersection of DEGs, WGCNA module genes (black + yellow), and LRGs in MSigDB. A total of six IS-associated hub genes, SLC2A3, NDUFB11, GTPBP3, SLC16A3, PUS1, and GRN, were obtained (Figure 4I). Additionally, the enrichment analysis of GO and KEGG of IS-related module genes found that GO enrichment results were mostly related to immune cells (Figure 5A), and KEGG enrichment results were also associated with immune signaling pathways. For example, T cell receptor signaling pathway, B cell receptor signaling pathway and cytokine– cytokine receptor interaction (Figure 5B). Finally, GSEA analysis of hub genes (Figure 5C) and prediction of upstream target miRNA (Figure 5D) were performed.

### 3.4 Subtype analysis of IS

**Figure 6:**
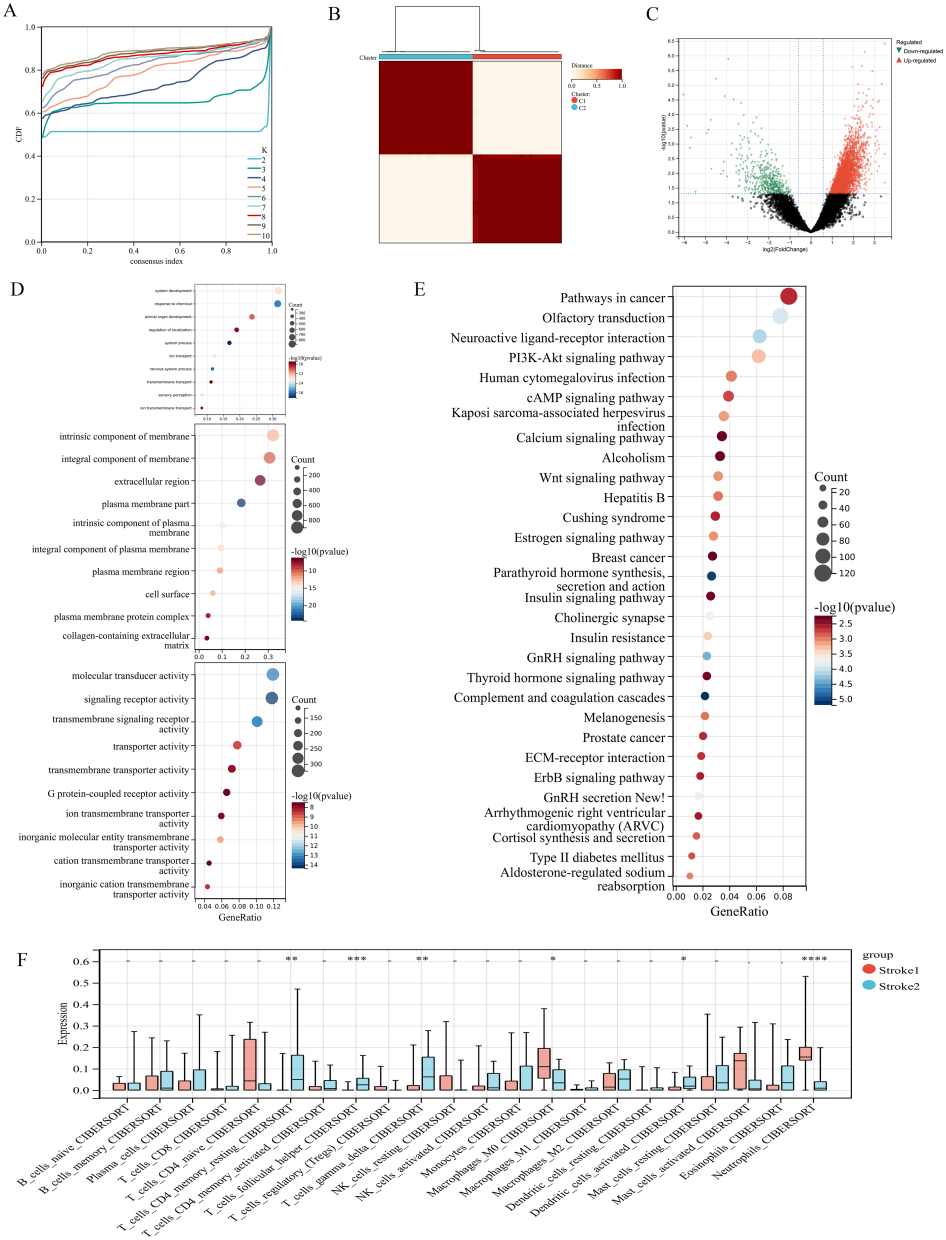
**(A)** The cumulative distribution function plot of cluster analysis. **(B)** Two subtypes of stroke samples were identified by consensus clustering analysis. **(C)** DEGs between two subtypes. **(D,E)** GO and KEGG enrichment analysis of DEGs among two subtypes, separately. **(F)** Differences in immune infiltration among different subtypes (CIBERSORT). *p < 0.05, **p < 0.01, ***p < 0.001, ****p < 0.0001.

The IS samples were classified into different subtypes based on the expression profiles of the six hub genes and ConsensusClusterPlus. The empirical cumulative distribution function plot shows that, when K = 2 (Figure 6A), delta has the smallest downward trend. Therefore, the samples were divided into two subtypes: stroke1 and stroke2 (Figure 6B). The DEGs of the two subtypes were screened with a threshold of |FC| > 1.5 and a *p*-value of < 0.05, and the results revealed 3,658 upregulated genes and 438 downregulated genes (Figure 6C). On the one hand, the results of enrichment analysis showed that the top 10 most significant results of GO BP included nervous system process, ion transport, and transmembrane transport, and the top 10 most significant results for CC include plasma membrane part, intrinsic component of plasma membrane, integral component of plasma membrane, and cell sur face, and the top 10 most significant results of MF include signaling receptor activity, transmembrane signaling receptor activity, and transmembrane signaling receptor activity and G protein-coupled receptor activity. On the other hand, the most significant results of KEGG enrichment analysis included complement and coagulation cascades, GnRH signaling pathway, neuroactive ligand–receptor interaction, in signaling pathways such as insulin resistance, PI3K–Akt signaling pathway, Wnt signaling pathway, and ECM–receptor interaction, and so on (Figures 6D,E). Finally, the results of immune infiltration between the subtypes showed similar results to those observed in the stroke group and the control group (Figure 6F). The neutrophil level was higher in stroke1 than in stroke2 across all the four algorithms, which further proves the key role of neutrophils in IS.

### 3.5 Prediction model and validation

**Figure 7:**
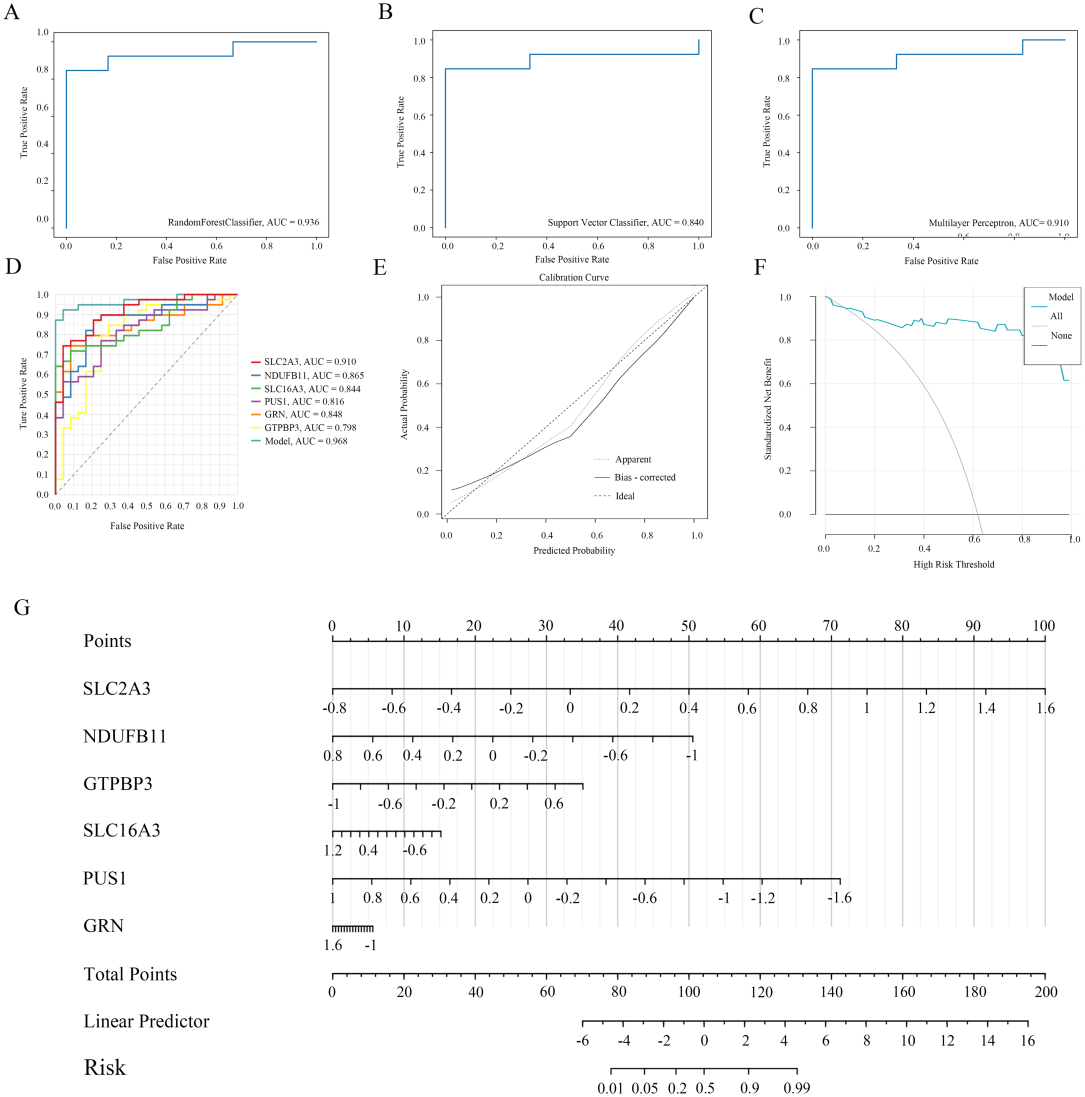
**(A)** The ROC area of diagnosis prediction model in RM. **(B)** The ROC area of diagnosis prediction model in SVM. **(C)** The ROC area of diagnosis prediction model in NN. **(D)** The ROC area of diagnosis prediction model in GLM. **(E)** The calibration curve of GLM. The confidence interval is 0.645, 95%CI [0.386–0.898]. **(F)** The decision curve of GLM. **(G)** The nomogram of GLM.

**Figure 8:**
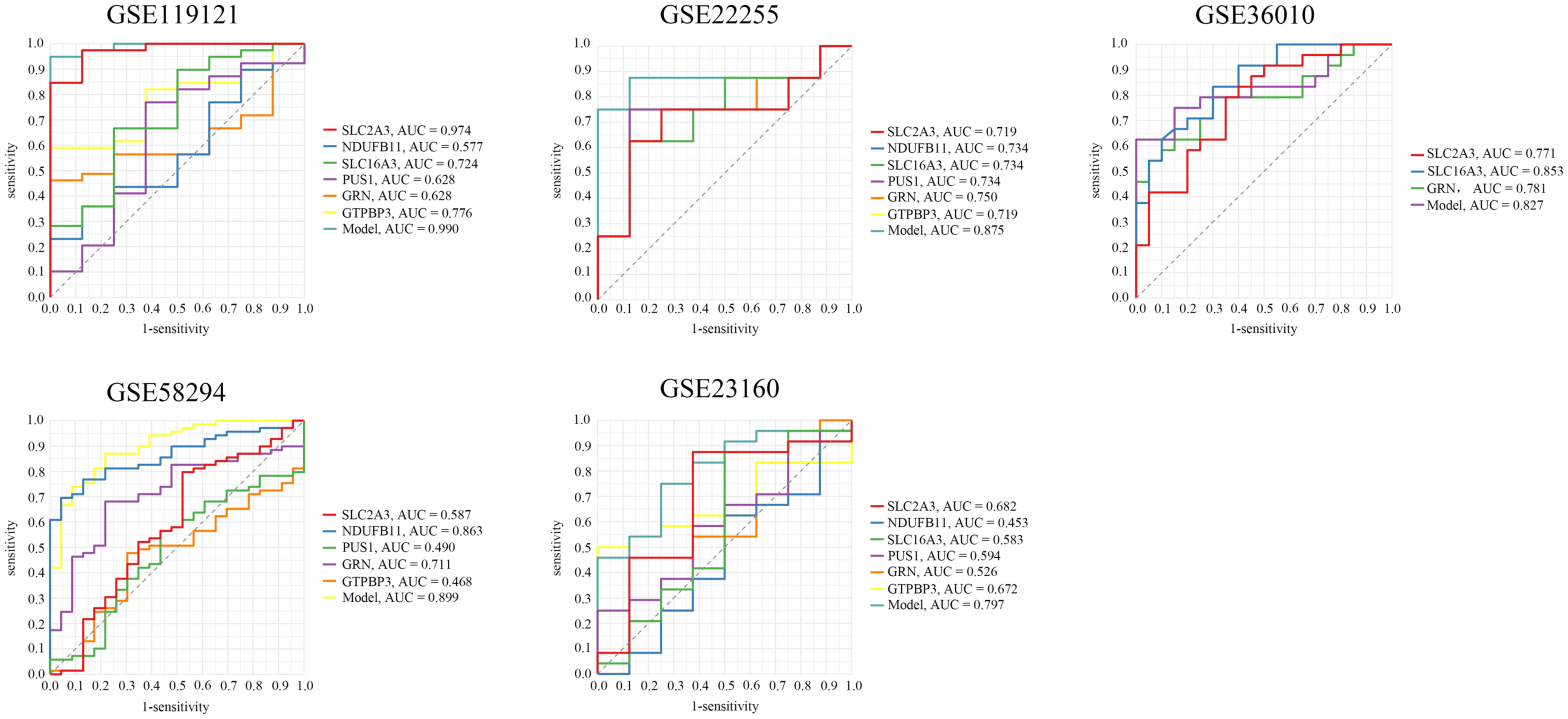
Validation of the GLM prediction model in external independent GEO datasets.

Given the lack of effective predictive biomarkers for IS, we constructed a diagnostic prediction model using six hub genes and four machine learning algorithms (including RM, SVM, NN, and GLM). The results showed that the AUC area of the RM model was 0.936, with a 95% CI of [0.931, 0.940] (Figure 7A). The AUC area of the SVM model was 0.840, with a 95% CI of [0.835, 0.844] (Figure 7B). The AUC area of the NN model was 0.910, with a 95% CI of [0.906, 0.915] (Figure 7C). In addition, the AUC area of the GLM model was 0.968 with a 95% CI of [0.928, 1] (Figure 7D), and the AUC of each hub genes were>0.75. Therefore, the GLM model—with the optimal predictive performance—was selected for further validation. A calibration curve was generated to evaluate the calibration performance of the model (Figure 7E), and a decision curve analysis and nomogram were also constructed (Figures 7F,G). The results show that the prediction performance of the model is excellent, judged by these results. To further verify the model’s generalizability, we conducted external validation by searching the GEO database for independent IS-related datasets. A total of five datasets were included for validation. Surprisingly, the AUC area of the ROC curves exceeded 0.75 in five of these datasets (Figure 8).

### 3.6 Relationship between hub genes and neutrophils

**Figure 9:**
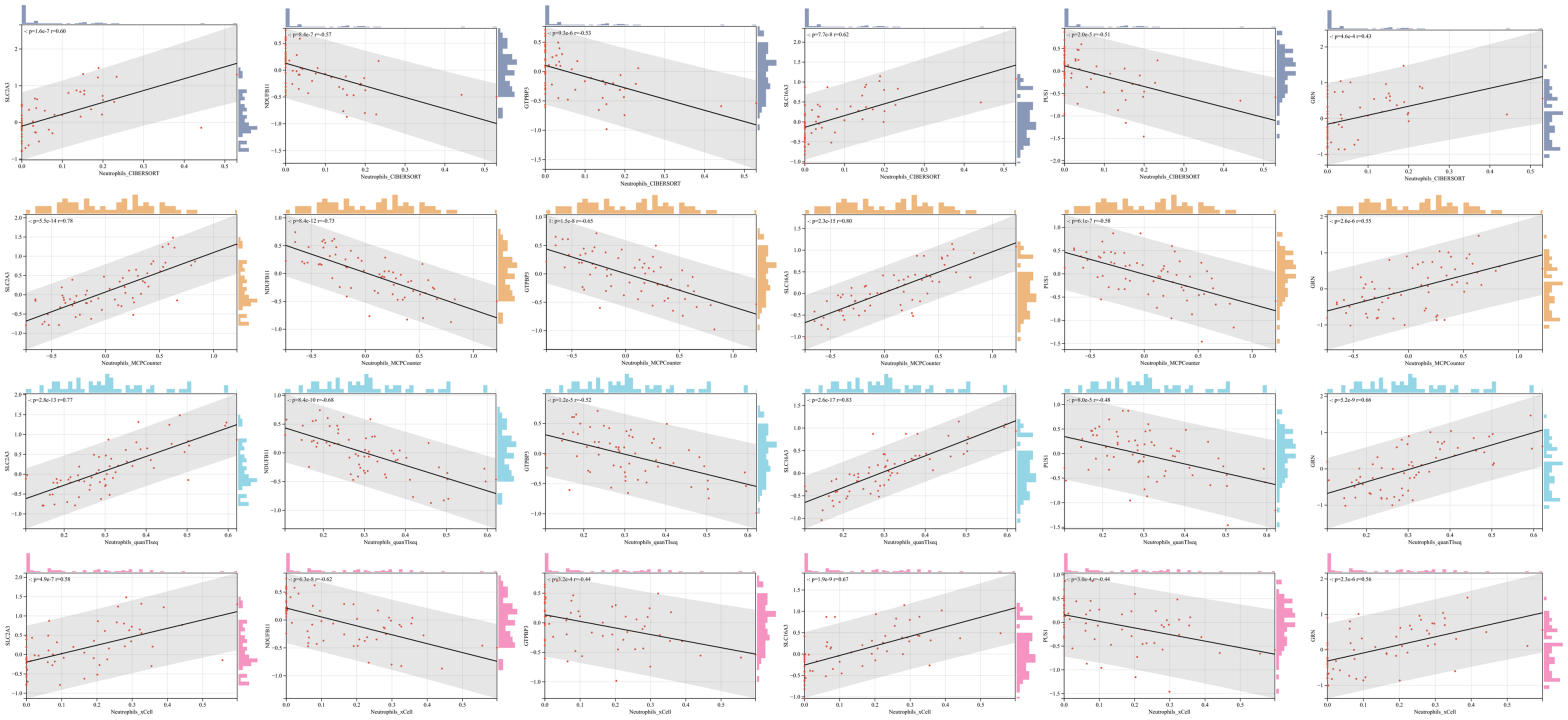
The correlation between six hub genes and neutrophils. *p* < 0.05 was considered statistically significant.

No matter the immune infiltration analysis of DEGs between the IS group and the control group, or in that between the stroke1 group and stroke2 subtypes, multiple immune infiltration algorithms showed statistical differences in neutrophils, and the most significant results of GO analysis of DEGs were mostly related to neutrophils. Therefore, it is reasonable to conclude that neutrophils play a crucial role in the occurrence and progression of IS. To further explore whether neutrophils are associated with the six hub genes, correlation analysis was conducted between each hub genes and the neutrophils infiltration scores derived from four immune infiltration algorithms. The results showed that all six hub genes were correlated with neutrophils across all four immune infiltration algorithms (Figure 9).

### 3.7 The expression of hub genes in OGD/R model

**Figure 10:**
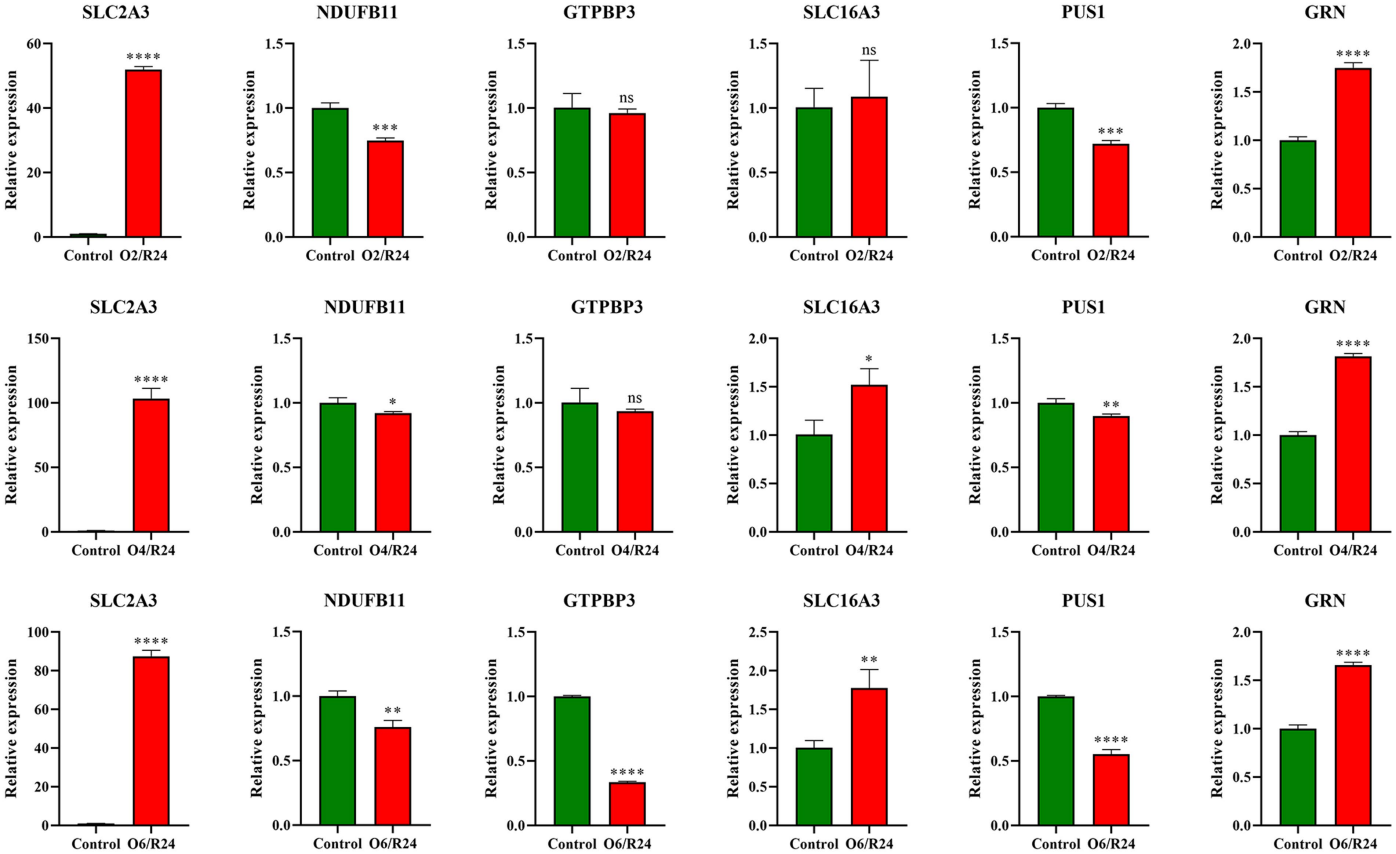
The expression level of hub genes in OGD/R model. The non-paired t-test was used for statistical analysis. O2/R24 (oxygen/glucose deprivation for 2 h and reperfusion for 24 h), O4/R24 (oxygen/glucose deprivation for 4 h and reperfusion for 24 h), O6/R24 (oxygen/glucose deprivation for 6 h and reperfusion for 24 h). *p < 0.05, **p < 0.01, ***p < 0.001, ****p < 0.0001.

**Figure 11:**
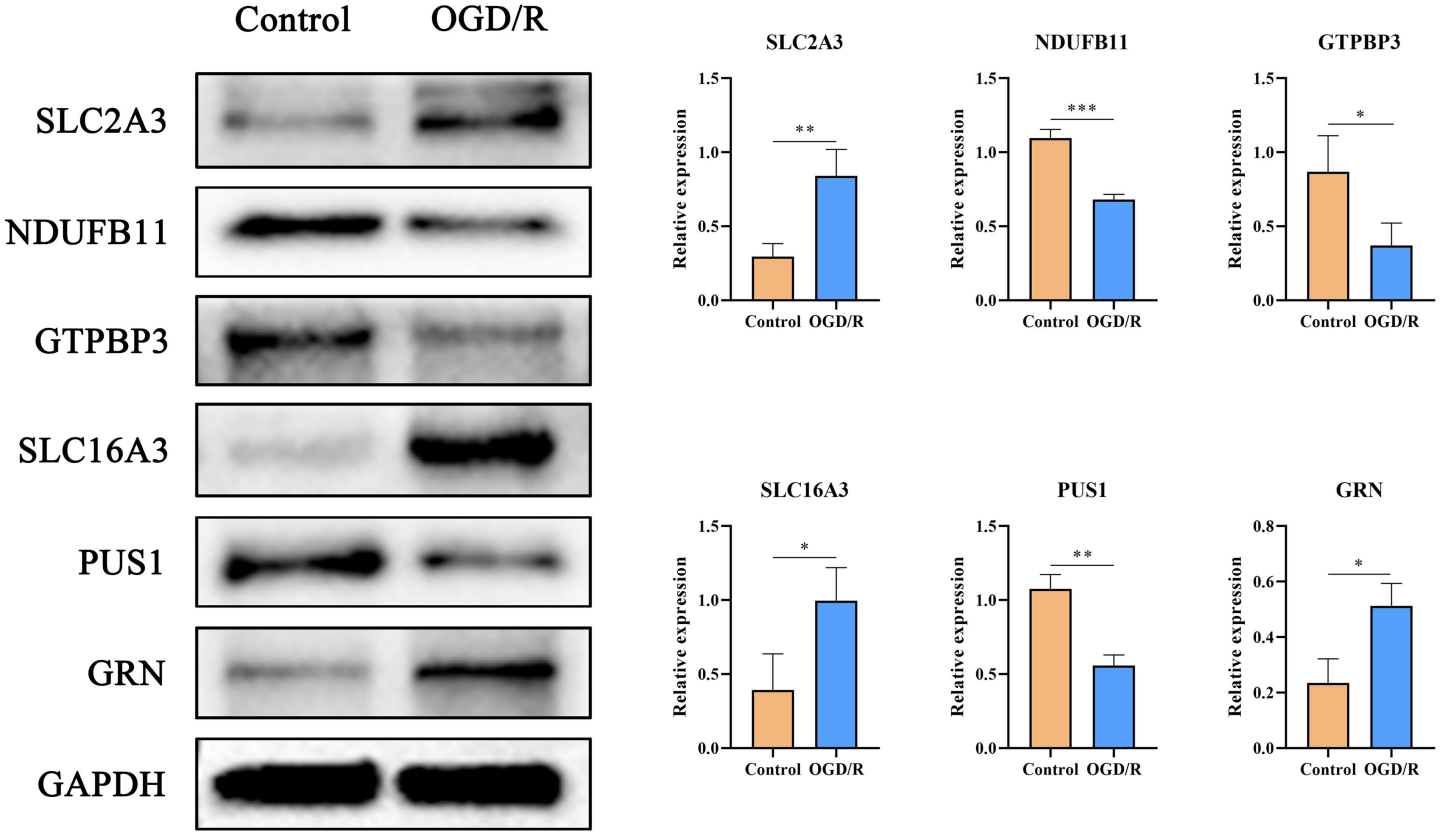
The protein expression levels of hub genes in OGD/R model. The non-paired t-test was used for statistical analysis. *p < 0.05, **p < 0.01, ***p < 0.001.

We detected the expression level of hub gene in the OGD/R model of PC12 neuronal cells. The results showed that the expression levels of SLC2A3, SLC16A3, and GRN in the OGD/R group were upregulated, while the expression levels of NDUFB11, GTPBP3, and PUS1 were downregulated. Although the expressions of GTPBP3 and SLC16A3 changed minimally in the early stage, they also showed the same trend. In addition, the results of WB showed similar trend with RT–qPCR. The above results are consistent with the trend of the GEO dataset GSE16561 (Figures 10, 11).

### 3.8 The effect of exogenous lactate on the proliferation of nerve cells induced by OGD/R

**Figure 12:**
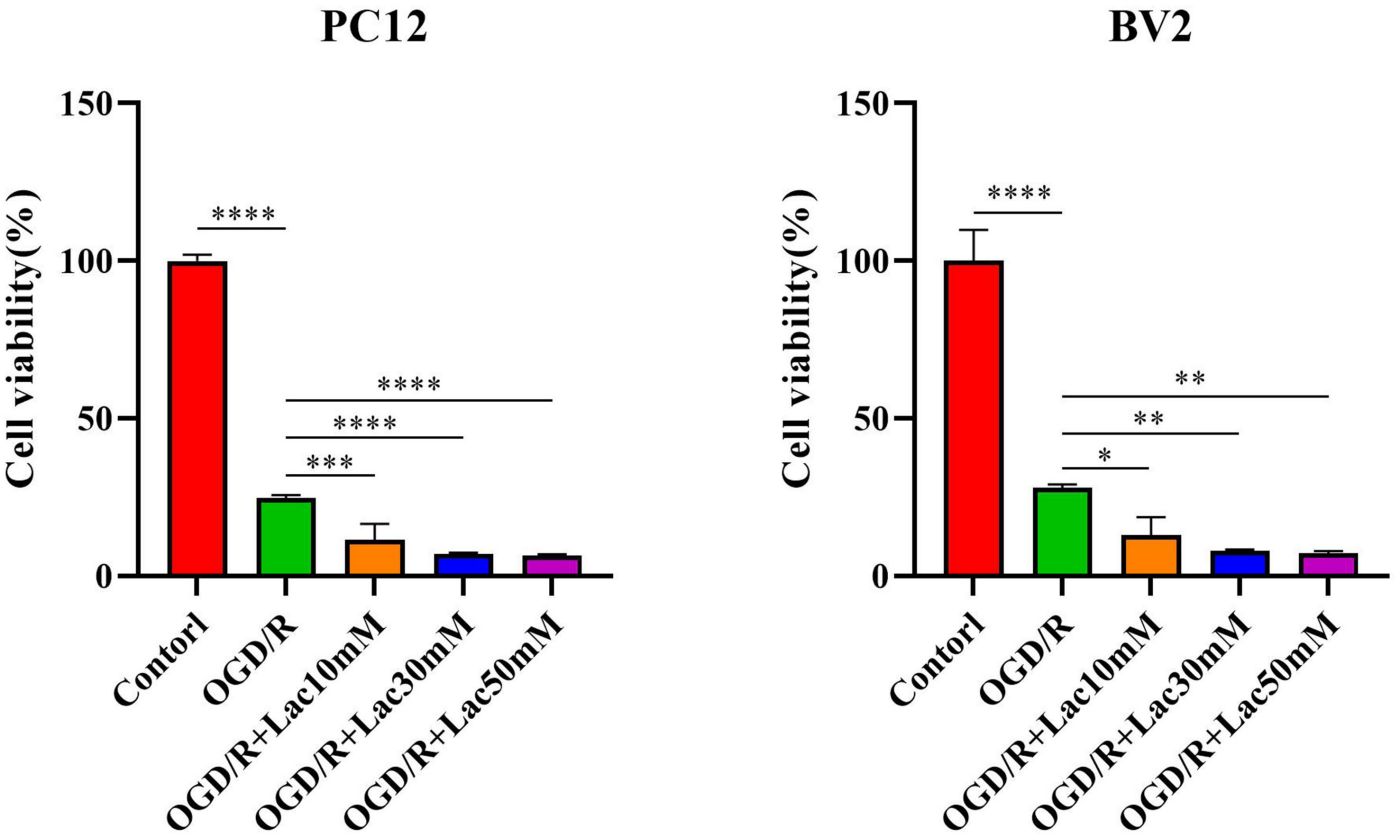
Exogenous lactate salt can further inhibit the proliferation of nerve cells after OGD/R. The differences among the groups were statistically analyzed using one-way ANOVA. The F/df value of ANOVA was *F* (4, 10) = 801.0. *p < 0.05, **p < 0.01, ***p < 0.001, ****p < 0.0001.

The results of the CCK8 assay showed that different concentration lactate could further inhibit the proliferation of neuronal cells following OGDR/R (Figure 12). This finding suggests that lactylation-related genes may be involved in regulating the occurrence and progression of IS by influencing the lactate level.

## 4 Discussion

The integration of artificial intelligence (AI) into clinical medicine has driven significant advancements in machine learning applications, particularly in disease diagnosis, prognostic prediction, and treatment strategy optimization through data-driven insights (Swanson et al., 2023). Notably, emerging paradigms employ machine learning to decipher epigenetic biomarkers, enabling precision medicine approaches for disease management and outcome prediction (Rauschert et al., 2020). In cerebrovascular research, these computational techniques have demonstrated clinical utility in multiple stroke-related domains, including IS diagnosis, the assessment of post-stroke cognitive impairment, and the prediction of mortality risk (Lee et al., 2023; Shurrab et al., 2024; Schwartz et al., 2023). However, despite these technological advancements, a critical gap persists in clinical translation of early diagnostic prediction models for stroke management. Building upon the established biological significance of protein lactylation in disease pathogenesis, our study systematically identified six lactylation-associated hub genes through integrative multidimensional analysis. These molecular biomarkers were subsequently utilized to develop a machine learning-powered diagnostic prediction model. The model’s robustness and generalizability were rigorously validated across multiple independent datasets, demonstrating consistent predictive performance through comprehensive computational validation frameworks.

Through integrative analysis intersecting DEGs, WGCNA modules, and LRGs, we identified six lactylation-associated hub genes implicated in IS pathogenesis: SLC2A3, NDUFB11, GTPBP3, SLC16A3, PUS1, and GRN. SLC2A3 is a glucose transporter critical for BBB function and cerebral glucose homeostasis (Yang et al., 2023). Experimental evidence indicates that SLC2A3 silencing suppresses glucose utilization and lactate generation, whereas its restoration enhances glycolytic activity and lactate production (Chen et al., 2018), and lactylation is induced by the glycolysis of glucose to produce lactate under hypoxia conditions (Jing et al., 2025). Our results indicate that the plasma membrane is significantly enriched in the cellular component (CC) analysis of GO; meanwhile, SLC2A3 is a lactate transporter located in the plasma membrane and its expression is upregulated in IS. Thus, our findings reveal the potential role of SLC2A3 in modulating lactylation via glucose transmembrane transport regulation, thereby contributing to IS pathophysiology. Furthermore, the family member of SLC2A3, GLUT1, has been demonstrated to be involved in the lactylation of glioblastoma and is regulated by glucose metabolism driven by PERK. TRIM29 is an upstream regulatory factor of PERK and can alleviate brain damage caused by ischemic stroke. Therefore, TRIM29 may control stroke by regulating PERK-mediated protein lactylation (De Leo et al., 2024; Wang et al., 2024; Deng et al., 2023). NDUFB11, a mitochondrial complex I subunit essential for electron transport chain integrity, maintains cellular energy metabolism and redox balance. Its deficiency correlates with impaired mitochondrial respiration, exacerbated oxidative stress, and apoptosis potentiation (Zhou et al., 2023). Clinically, NDUFB11 down-expression associates with atherosclerotic progression and adverse cardiovascular outcomes (Yang et al., 2023), which is particularly relevant given that 20% of IS originates from atherosclerotic plaque rupture and subsequent thromboembolism (Puig et al., 2023). The observed NDUFB11 downregulation in our IS cohort implies dual pathogenic mechanisms: mitochondrial dysfunction potentiation and atherosclerosis acceleration. GTPBP3, a mitochondrial GTPase involved in tRNA modification, exhibits functional convergence with NDUFB11 in respiratory chain regulation. Depletion of GTPBP3 leads to respiratory defects, impaired mitochondrial translation, and lactic acidosis (Kopajtich et al., 2014; Asano et al., 2018). SLC16A3 (MCT 4) mediates lactate efflux to sustain glycolytic flux. Tumor models demonstrate that SLC16A3 overexpression enhances lactate export, while its inhibition suppresses the Warburg metabolism (Yu et al., 2024). In addition, the concept of lactate shuttle as a means of distributing potential energy and providing reduction–oxidation signaling mechanisms within and between cells has been recognized in the field of neuroscience. In simple terms, lactate produced within glial cells is released by SLC16A3 outside the cell to be taken up by neighboring neurons (Roumes et al., 2021). Experimental studies reveal PPARα agonism upregulates SLC16A13, augmenting myocardial lactate levels as potential lactylation substrates (Silagi et al., 2020; Sun et al., 2024). Our observed SLC16A3 upregulation may suggest its involvement in ischemic lactate overproduction and subsequent lactylation cascades, and another possibility is that hypoxic conditions induce the compensatory upregulation of SLC16A3 expression, thereby enhancing lactate biosynthesis as an adaptive energy substrate, and this metabolic adaptation facilitates cellular energy homeostasis through intercellular lactate shuttling. PUS1 deficiency links to mitochondrial myopathy with lactic acidosis via disrupted oxidative phosphorylation (Zeharia et al., 2005). As a ubiquitin-binding protein, PUS1 additionally stabilizes polyubiquitinated substrates against deubiquitinase activity (Hartmann-Petersen et al., 2003). The observed PUS1 downregulation may thus exacerbate ischemic injury through dual mechanisms: lactic acid accumulation and impaired protein homeostasis. GRN, a pleiotropic growth factor regulating neuroinflammation and neuronal survival, demonstrates genetic associations with neurodegenerative disorders and neuroinflammatory disease (Kleinberger et al., 2013). In conclusion, the six hub genes may be involved in the occurrence and development of IS by regulating the mitochondrial respiratory chain, glycolysis, lactate transmembrane transport, and so on and may be key biomarkers for predicting prognosis.

IS not only triggers localized neuroinflammation but also induces systemic immunomodulatory effects, forming a complex neuroimmune axis that influences disease progression. The therapeutic targeting of neuroinflammatory cascades has long been a focus in stroke research (Simats and Liesz, 2022). Neuroinflammation in IS manifests through dual mechanisms: (1) activation of microglia/astrocytes and recruitment of peripheral immune cells and (2) overproduction of pro-inflammatory mediators (e.g., cytokines, chemokines, and ROS) that collectively compromise BBB integrity and exacerbate neuronal injury (Alsbrook et al., 2023). Our multi-modal analysis revealed significant immune pathway enrichment across both pan-transcriptomic and module-specific DEGs, and several immune cells are upregulated in IS, such as monocytic lineage, neutrophils, T-cell gamma delta, microphages M0, microphages M1, and mast cells. Strikingly, neutrophils were the only immune cell type showing statistically significant elevation across all computational methods, positioning it as central mediators of post-stroke inflammation. Previous studies have shown that neutrophils serve a complex function as a precursor to brain damage after IS (Li et al., 2022). Increasing evidences identify neutrophil-derived extracellular traps (NETs) as dual-edged effectors: While antimicrobial through pathogen entrapment, their excessive formation promotes microvascular thrombosis and worsens clinical outcomes (Denorme et al., 2022). In addition, neutrophils are closely related to lactyaltion. Hypoxia drives CD71 + neutrophil glycolysis, generating lactate that facilitates histone lactylation—an epigenetic mechanism implicated in tumor immunomodulation (Ugolini et al., 2025). HMGB1 lactation induces acute kidney injury in sepsis by driving the formation of NETs (Zhu et al., 2024). Series of evidences demonstrate that lactylation is involved in the regulation of neutrophils. Our integrative analysis revealed significant correlations between the six hub genes and neutrophil infiltration scores across four independent immunophenotyping algorithms. These findings suggest that SLC2A3, NDUFB11, GTPBP3, SLC16A3, PUS1, and GRN may collectively modulate IS-associated neuroinflammation through neutrophil-mediated mechanisms. Mechanistically, KEGG pathway enrichment analysis of both pan-transcriptomic DEGs and WGCNA module genes implicated T cell receptor signaling pathway as a potential regulatory interface bridging lactylation dynamics and neutrophil biology. In addition, we revealed the difference between the two stroke subtypes distinguished by hub genes and the possible signaling pathways involved. We found that the expression level of neutrophils in stroke1 was higher than stroke2, while SLC2A3 and SLC16A3 were higher in stroke1 than stroke2, and GRN was lower in stroke1 than stroke2. Therefore, SLC2A3, SLC16A3, and GRN are more likely to regulate the infiltration level of neutrophils in IS and neuroinflammation through lactylation.

To investigate more accurate early diagnostic models for IS, we employed six hub genes to construct predictive models using four machine learning algorithms. Internal validation demonstrated robust performance across all models, with AUC values exceeding 0.75, indicative of excellent predictive capability. The GLM, exhibiting the highest AUC, was selected to develop a nomogram and calibration curve. To further validate the model’s generalizability, external validation was performed using multiple IS-related datasets from the GEO database. Remarkably, the model consistently achieved strong predictive performance across all external cohorts. These results highlight the clinical utility of this model as a reliable tool to enhance diagnostic precision in IS management. Besides, lactylation is closely related to the level of lactate. To explore the possible mechanism by which LRGs regulate IS, we found that exogenous lactate salt could further inhibit the proliferation of neuronal cells following OGD/R. This might be related to key hub genes. Finally, studies have shown that the predictive model that combines conventional MR images and clinical variables available during the early acute stage of stroke can predict the clinical outcomes 90 days after stroke. Moreover, this study emphasize that the integrated clinical and imaging model is superior to models that include either one or the other method (Liu et al., 2023). Therefore, it is advisable to consider integrating our model with MRI to enhance the predictive performance in the future.

## 5 Conclusion

Lactylation has been increasingly recognized as a critical regulatory mechanism in the pathogenesis of various diseases. In this study, we identified six lactylation-associated hub genes via integrative analysis of DEGs, WGCNA, and LRGs. We also systematically characterized the immune microenvironment of IS using multiple immune infiltration scoring algorithms—among which neutrophils emerged as key pathogenic contributors. Four machine learning algorithms were used to build predicted models based on the six hub genes. The GLM, exhibiting the highest AUC, was selected for nomogram construction and showed excellent prediction effect. External validation confirmed the model’s generalizability. This study establishes a novel diagnostic framework for IS and advances mechanistic understanding of lactylation-mediated neuroinflammatory pathways.

## Data Availability

The datasets presented in this study can be found in online repositories. The names of the repository/repositories and accession number(s) can be found in the article/supplementary material.

## Footnotes

^1^ https://www.ncbi.nlm.nih.gov/

^2^ https://www.gsea-msigdb.org/gsea/msigdb/

^3^ http://software.broadinstitute.org/gsea/index.jsp

^4^ http://www.gsea-msigdb.org/gsea/downloads.jsp

^5^ https://www.targetscan.org

## Glossary

IS: Ischemia stroke
GEO: Gene Expression Omnibus
WGCNA: Weighted Gene Co - expression Network Analysis
RM: Random Forest
SVM: Support Vector Machine
NN: Neural Network
GLM: Generalized Linear Models
BBB: Blood - brain barrier
DEGs: Differentially expressed genes
LRGs: Lactylation-related genes
GSEA: Gene Set Enrichment Analysis
MM: Module Membership
GS: Gene Significanc
GO: Gene Ontology
KEGG: Kyoto Encyclopedia of Genes and Genomes
FC: Fold change
BP: Biological Process
CC: Cellular Component
MF: Molecular Function
OGD/R: Oxygen–Glucose Deprivation/Recovery
